# Cardiovascular Changes in Women Undergoing Medicated and Natural Frozen Embryo Transfer Cycles: A Prospective Observational Cohort Study

**DOI:** 10.3390/jcm15124717

**Published:** 2026-06-17

**Authors:** Freya Baird, Eleni Kakouri, Iulia Huluță, Ippokratis Sarris, Kypros H. Nicolaides, Nikos A. Kametas

**Affiliations:** 1 King’s Fertility, Fetal Medicine Research Institute, 16-20 Windsor Walk, Denmark Hill, London SE5 8BB, UK; 2 Department of Women’s Health, Faculty of Life Sciences and Medicine, King’s College London, Great Maze Pond, London SE1 9RT, UKnick.kametas@kcl.ac.uk (N.A.K.); 3 Fetal Medicine Research Institute, 16-20 Windsor Walk, Denmark Hill, London SE5 8BB, UK

**Keywords:** assisted reproductive technology, frozen embryo transfer, medicated cycle, natural cycle, maternal circulation, echocardiography, cardiovascular changes

## Abstract

**Background:** Frozen embryo transfer (FET) use in assisted reproductive technology (ART) has increased globally, with multiple reviews linking FET—particularly medicated cycles—to higher risks of obstetric complications including hypertensive disorders of pregnancy (HDP). Given that HDP is a sex-specific risk factor for future cardiovascular disease (CVD), this study aimed to assess acute cardiovascular changes in medicated versus natural modified FET cycles. **Methods:** This was a prospective observational cohort study at a fertility centre in London. Patients were recruited from May 2021 to March 2022. Maternal demographics including age, body mass index, smoking status, ethnicity and parity were recorded. Cardiovascular parameters including blood pressure along with measures of left ventricular systolic and diastolic function, assessed by transthoracic echo, were analysed at baseline in the luteal phase of the preceding cycle and on the day of embryo transfer, in medicated and natural modified FET cycles. Repeat measures analysis of the cardiac variables for the two time points, comparing the two protocols after controlling for maternal demographics, was performed by linear mixed models. **Results:** Seventy-two healthy patients were included in the analysis; of those, 59 (82%) underwent the medicated protocol. For both protocols, after controlling for maternal demographic characteristics, the left atrial area significantly increased (*p* = 0.004) from baseline to embryo transfer with a mean difference of 0.98 (95% CI [0.33, 1.63]). When comparing the interaction between the protocols between the two time points, whilst no effect could be seen on haemodynamic variables or left ventricular diastolic/systolic function, medicated FET cycles were associated with a statistically significant improvement in mean average global longitudinal strain (GLS) (*p* = 0.024) with a mean difference of −2.24 (95% CI [−4.17, −0.31]), whereas natural modified cycles demonstrated a slight shift toward more positive strain values. **Conclusions:** In this cohort of healthy patients undergoing FET, both protocols were associated with a significant increase in left atrial area from baseline to embryo transfer possibly resulting from an increased preload due to progesterone administration. The improvement in left ventricular average GLS seen in medicated FET cycles may reflect protocol-related physiological effects, potentially mediated by sustained exogenous oestrogen exposure and its influence on vascular loading conditions and myocardial relaxation.

## 1. Introduction

Globally, the trend towards frozen embryo transfer (FET) cycles within assisted reproductive technology (ART) has increased. Preliminary figures from the Human Fertilisation and Embryology Authority (HFEA) quoted an increase from around 20% in 2013 to 39% in 2023 in the UK [1]. Figures from the European IVF Monitoring Consortium (EIM) for the European Society of Human Reproduction and Embryology (ESHRE) have quoted over 1 million ART treatment cycles performed in 2019, of which 335,744 were frozen embryo transfer cycles [2]. Advancements in controlled ovarian hyperstimulation (COH), facilitating the creation of multiple embryos per stimulation cycle, along with improved cryopreservation techniques, have allowed for single embryo transfers within FET cycles to mitigate the risk of multiple gestations. In addition, the incorporation of screening tools such as pre-implantation genetic testing (PGT) into fresh IVF cycles are just a few factors that have contributed to the rise in FET cycles worldwide.

It has been widely reported that there is an increased association between the use of ART and subsequent maternal and perinatal complications in pregnancy resultant from fresh and frozen cycles [3]. In more recent years, the focus has been on the increased risk associated with frozen embryo transfer (FET) cycles when compared to fresh cycles [4,5] and more specifically the increased risk with medicated FET cycles when compared to natural/natural modified (NM) FET cycles [6,7,8,9,10]. The obstetric risks include hypertensive disorders of pregnancy (HDP), which are recognised as a sex-specific risk factor for the development of cardiovascular disease and are coded as such in cardiovascular and obstetric society guidelines [11].

There is ongoing debate as to whether the underlying cause for such risks is due to the fertility treatment and protocol implemented, the underlying cause of infertility including maternal age, or possible pre-existing co-morbidities. Reassuringly, whilst initial studies suggested a link between ART and the risk of cardiovascular disease (CVD), in particular, the risk of stroke [12], subsequent national-level studies and meta-analyses did not find an association between ART and major adverse cardiovascular outcomes [13,14]. The findings suggest that while ART may be associated with an increased risk of adverse obstetric outcomes, the relationship between ART and maternal cardiovascular health remains complex and not fully understood. Little is known about the effect of IVF on the cardiovascular system. A recent systematic review and meta-analysis [15] examining acute haemodynamic changes during fresh IVF cycles, mostly focusing on long down-regulation agonist protocols with limited analysis of cardiac function, demonstrated heterogeneity within the results. Another more recent study observed haemodynamic changes within fresh IVF cycles in the short, antagonist protocol [16]. Of the 68 patients analysed, a significant increase in cardiac output (CO) and stroke volume index (SVI) at peak oestradiol levels was noted when compared to baseline, along with a significant decrease in systemic vascular resistance index (SVRI). Despite this contribution to the literature, given the increased risks noted in frozen embryo transfer cycles, to date no study has assessed cardiac function during FET cycles.

The aim of this study is to compare cardiac function at baseline and then at the point of embryo transfer between medicated and natural/natural modified FET cycles.

## 2. Materials and Methods

### 2.1. Study Design, Population and Demographics

**Figure 1:**
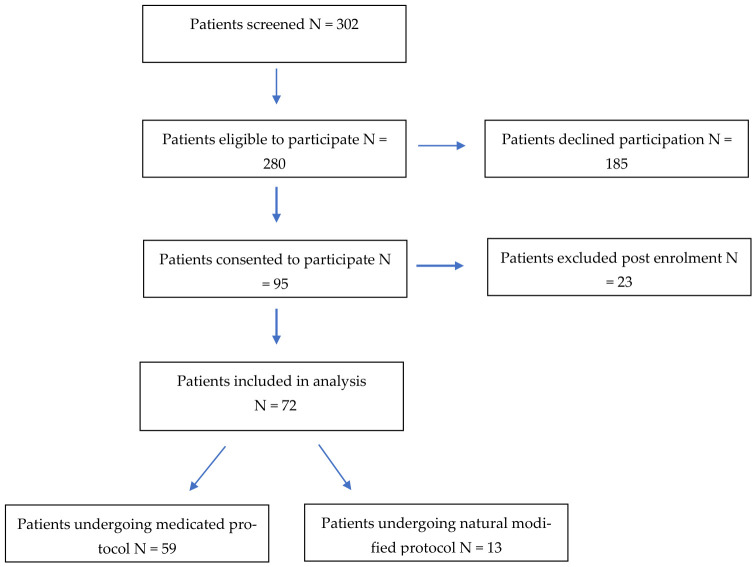
Flow chart demonstrating patient recruitment pathway.

This was a prospective observational cohort study carried out in collaboration with King’s Fertility and the Fetal Medicine Research Institute in London, UK. Local ethics approval was granted by an ethics committee: Wales REC 7 20/W A/03233. Patients were enrolled into the study following written consent between May 2021 and March 2022 before the commencement of their treatment cycle. Patient recruitment is shown in Figure 1. Patients deemed eligible to participate included women over 18 years of age who were due to start their FET cycle with either a medicated or natural modified cycle. Exclusion criteria were cardiac, renal, or liver disease and diabetes. Participants’ electronic patient records, stored on the IDEAS software package, Version 7 (Mellowood Medical Inc., Toronto, ON, Cananda), were reviewed to establish background medical history.

A medicated FET cycle was defined as follows: exogenous oestrogen in the form of estradiol valerate, administered from day 2 of the menstrual cycle at a dose between 6 and 8 mg per day +/− the addition of estradiol 100 mcg transdermal patches if clinically required aiming for an endometrial thickness of >/=7 mm, followed by the additional of vaginal progesterone pessaries: lutigest at 100 mg 3 times/day, 5 days prior to embryo transfer. Natural modified cycles were defined as protocols utilising a human chorionic gonadotropin (hCG) trigger when a dominant follicle > 14 mm was noted with an endometrial thickness >/= 7 mm, followed by vaginal progesterone pessaries 2 days later for 5 days prior to embryo transfer. Protocol selection was guided by a combination of patient preference and clinician judgement. At the time of the study, the most commonly performed protocol was the medicated FET.

Baseline demographics were assessed at the baseline study visit and included age, height and weight, ethnicity (self-reported: White, Black, South Asian, East Asian and other), smoking status (yes or no), menstrual cycle regularity (24–38 days) [17] and parity.

### 2.2. Blood Pressure and Transthoracic Echocardiography

Blood pressure (BP) was measured using a mercury sphygmomanometer (Accoson Dekamet, AC Cossor & Son (Surgical) Ltd., London, UK) in accordance with the British Hypertension Society [18]. Mean arterial pressure (MAP) was derived using the conventional equation, MAP = (SBP + 2 × DBP)/3, with SBP and DBP representing systolic and diastolic blood pressure values, respectively.

Comprehensive cardiac evaluation was conducted using two-dimensional (2D), M-mode, power-wave and colour Doppler, and speckle-tracking echocardiography (STE) employing a 3.5 MHz transducer (Toshiba Aplio CV; Toshiba Corporation, Tokyo, Japan), following the recommendations of the American Society of Echocardiography [19]. Further description outlining the echocardiography assessment can be found as previously described in our earlier study [20] and presented here in Supplementary Material S1 [21].

The measurements were performed at 2 time points: Visit 1, baseline scan performed in the luteal phase of the menstrual cycle directly preceding the treatment cycle and Visit 2, day 5 embryo transfer (day 5/6 blastocyst transfer).

### 2.3. Statistical Analysis

The Kolmogorov–Smirnov test was used to assess the normality of the distribution of the data. As all examined numerical variables were normally distributed, descriptive analysis was performed using mean +/− standard deviation for quantitative variables, and n (%) for categorical variables. Differences in demographics between the two study groups were compared with the chi-squared test or Fisher’s exact test for categorical data and independent *t*-tests for numerical data.

**Figure 2:**
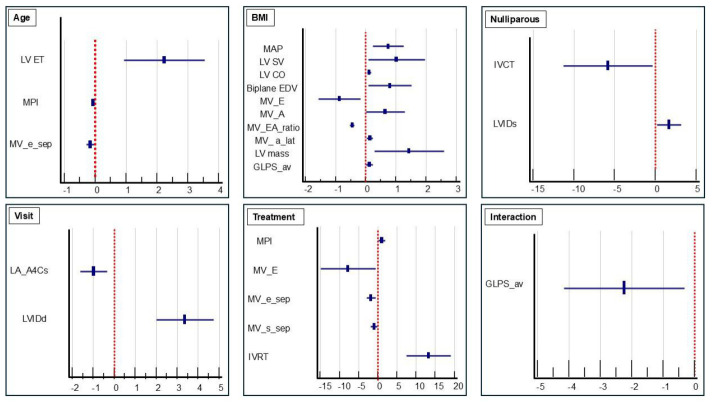
Forest plot diagrams for the multilevel linear mixed-effects models for cardiovascular parameters. The figure demonstrates the coefficients (95% CI) for the fixed effects of the final model including only statistically significant variables. The initial model with all variables is presented in Supplementary Table S2 and the corresponding data for the current figure can be found in Supplementary Table S3. LV ET—left ventricular ejection time, MPI—myocardial performance index, MV_e_sep—mitral valve e-septal velocity, MAP—mean arterial pressure, LV SV—left ventricular stroke volume, LV CO—left ventricular cardiac output, EDV—end diastolic volume, MV_E—mitral valve E-wave maximum velocity, MV_A—mitral valve A-wave maximum velocity, MV_EA_ratio—mitral valve E/A-wave ratio, MV_a_lat—mitral valve a-lateral velocity, LV mass—left ventricular mass, GLPS_av—average global longitudinal strain, IVCT—Isovolumic contraction time, LVIDs—left ventricular end-systolic diameter, LA_A4Cs—left atrial area, LVIDd—left ventricular end-diastolic diameter, MV_s_sep—mitral valve s-septal velocity, IVRT—isovolumic relaxation time.

**Figure 3:**
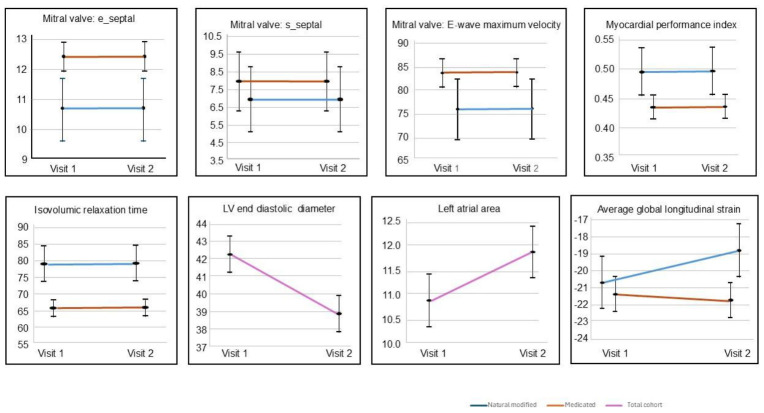
Longitudinal changes in cardiovascular parameters that were statistically significantly different between medicated (orange lines) and natural modified (blue lines) participants between Visit 1 (baseline) and Visit 2 (at embryo transfer). In purple lines, we depict changes over time in the two variables that changed in a similar way, with no difference between the 2 groups. Data show the estimated marginal means from the linear mixed model analysis, where the estimated value is provided after controlling for other predictors.

A multilevel linear mixed-effects model was used for the repeated measures analysis of the cardiac variables for the two time points, comparing the two protocols after controlling for maternal demographics. We controlled for age, body mass index, ethnicity (White, Black, South Asian, East Asian, other), smoking (yes/no), and nulliparity (yes/no). The fixed-effect component included time (the two visits), study group (medicated versus natural modified FET), age, body mass index, ethnicity, smoking, nulliparity, and first-order interaction between time and group. The likelihood ratio (LR) test was used to define the best multilevel model (including only the random slope for time or random intercept versus including both the random intercept and slope) and to compare it with the base model (with no random effects). The results of multilevel mixed-effects models are presented in Figure 2 and Supplementary Tables S2 and S3. The estimated marginal means of the significantly different variables between groups at each group/time combination are presented in Figure 3. In Figure 2, we present only significant variables remaining in the final model and in Supplementary Table S2 all the variables included in the model in their initial analysis. Supplementary Table S3 includes the corresponding data as presented in Figure 2. Due to the large volume of data, we present the fixed and not the random effects of the model.

Data were analysed using the statistical software package SPSS (released 2010, IBM SPSS Statistics for Windows, Version 31.0, IBM Corporation, Armonk, NY, USA). A *p*-value < 0.05 was considered statistically significant. 

## 3. Results

Of the patients who were approached, 95 patients consented to participate in the study and to be prospectively followed between Visit 1 and Visit 2. None of the patients approached had cardiac, renal, liver disease, or diabetes. In total, 72 patients completed the study measurements for both time points and were included in the final analysis, including 59 patients undergoing a medicated FET cycle, and 13 undergoing a natural/modified FET cycle. The recruitment process is summarised in Figure 1 and was carried out in parallel with our first published study assessing for cardiac changes in patients with and without previous ART treatment [20].

### 3.1. Demographic Characteristics

A summary of maternal demographic characteristics for the full cohort, and their comparison across the two groups, is provided in Table 1. The mean age of participants was 36.3 ± 3.6 years, with no significant difference between the NM FET (37.2 ± 2.7) and medicated FET (36.1 ± 3.8) groups (*p* = 0.378). Height, weight, BMI, and body surface area were similar between groups (all *p* > 0.2). Ethnicity differed significantly between groups (*p* = 0.038), with a higher proportion of White participants in the NM FET group (92.3% vs. 61.0%), whereas Black, South Asian, and other ethnicities were represented only in the medicated FET group. Smoking status, regular menstrual cycles, and nulliparity were comparable between groups (all *p* ≥ 0.494).

**Table 1:** Study population baseline demographic characteristics are presented for the total population and sub-categorised according to treatment protocol.

Variable	Total Population (*n* = 72)	Natural Modified FET (*n* = 13)	Medicated FET (*n* = 59)	*p*-Value
**Baseline characteristics (mean ± SD/*n* (%))**				
Age (years)	36.3 ± 3.6	37.2 ± 2.7	36.1 ± 3.8	0.378
Height (cm)	164.5 ± 6.8	166.6 ± 5.4	164.0 ± 7.1	0.220
Weight (kg)	65.9 ± 11.0	64.9 ± 7.4	66.1 ± 11.7	0.712
Body Mass Index (kg/m^2^)	24.4 ± 4.0	23.3± 2.3	24.6 ± 4.3	0.376
Body Surface Area (m^2^)	1.72 ± 0.14	1.72 ± 0.11	1.72 ± 0.15	0.834
Ethnicity				0.038
White, *n* (%)	48 (66.7)	12 (92.3)	36 (61.0)	
Black, *n* (%)	8 (11.1)	0 (0.0)	8 (13.6)	
South Asian, *n* (%)	10 (13.9)	0 (0.0)	10 (16.9)	
East Asian, *n* (%)	1 (1.4)	1 (7.7)	0 (0.0)	
Other, *n* (%)	5 (6.9)	0 (0.0)	5 (8.5)	
Smoking, *n* (%)	1 (1.4)	0 (0.0)	1 (1.7)	1.000
Regular menstrual cycles, *n* (%)	68 (94.4)	13 (100)	55 (93.2)	1.000
Nulliparous, *n* (%)	52 (72.2)	8 (61.5)	44 (74.6)	0.494

FET: frozen embryo transfer, SD: standard deviation.

### 3.2. Cardiovascular Variables

Comparisons of haemodynamic and cardiac variables at baseline for the total population and across the two groups are presented in Supplementary Table S1. In brief, all subjects had normal cardiac measurements. When unadjusted values were compared between the two groups, mitral valve e-septal velocity was higher and left ventricular isovolumic relaxation time was lower in the medicated FET, compared to the NM FET group.

The *p*-values for the variables assessed against all demographics in the initial model can be found in Supplementary Table S2. For the final model, when assessing firstly the haemodynamic variables, BMI was the only demographic variable to show a statistically significant result against MAP (*p* = 0.005), left ventricular (LV) stroke volume (*p* = 0.034), and LV cardiac output (*p* < 0.001). When reviewing LV systolic function, again BMI was statistically significant for biplane LV end-diastolic volume (*p* = 0.02). The variable time (Visit 2 versus 1) was the parameter found to be statistically significant for left atrial area (0.004). Left atrial area was significantly higher at embryo transfer with a mean difference of 0.98 (95% CI [0.33, 1.63]).

Age was statistically significant for the LV ejection time (*p* = 0.001). Age was also found to be statistically significant for the myocardial performance index (*p* = 0.023) along with the intended treatment (*p* = 0.003). Specifically, MPI was found to be higher in the natural modified group when compared to the medicated group with a mean difference of 0.064 units (95% CI 0.022–0.107). Nulliparity was statistically significant for the LV isovolumic contraction time (*p* = 0.037).

Analysing LV diastolic function, BMI, again was found to be statistically significant for the following: mitral valve A-wave maximum velocity (*p* = 0.046), mitral valve E/A-wave ratio (*p* = 0.002) and mitral valve a-lateral velocity (*p* = 0.013). For the mitral valve E-wave maximum velocity, both BMI (*p* = 0.015) and the intended treatment (*p* = 0.034) (Figure 3) were considered statistically significant parameters. Mitral valve E-wave velocity was lower in the natural modified group when compared to the medicated group with a mean difference of −7.76 cm/s (95% CI −14.91–−0.60) (Figure 3). The intended treatment was also found to be statistically significant for mitral valve s-septal velocity (*p* = 0.035) and mitral valve e-septal velocity ((*p* = 0.003) (Figure 2), along with age (*p* = 0.013)). The intended treatment was also found to be significant for the isovolumic relaxation time (*p* <0.001) (Figure 3).

Assessing LV m-mode, BMI was statistically significant for LV mass (*p* = 0.015), nulliparity for LV end-systolic diameter (0.024), and the visit was again found to be statistically significant for end-diastolic diameter which was significantly smaller at embryo transfer compared to baseline with a mean difference of −3.37 mm (95% CI–−4.73–−2.01) (*p* ≤ 0.001) (Figure 3).

The last analysis focused on global strain. The average global longitudinal strain was statistically affected by both BMI (*p* = 0.041) and the interaction between the treatment and visit (*p* = 0.024) (Figure 3). A full summary of all the coefficients for the final model can be found in Figure 2 and Supplementary Table S3.

## 4. Discussion

### 4.1. Main Findings and Interpretation

This is the first study, to our knowledge, to investigate changes in cardiac function in women during frozen embryo transfer cycles in both medicated and natural modified protocols.

Our findings correlate with past studies that have associated BMI with changes in cardiac function [22,23], with improvements in BMI also leading to positive effects [24]. Age was also found to independently affect cardiovascular parameters as expected, having been widely acknowledged in the literature [25]. The linear mixed model showed that parity significantly predicted IVCT. Parous women had significantly lower IVCT values compared with nulliparous women.

Pregnancy is associated with substantial cardiovascular adaptations, including increased blood volume, ventricular remodelling and reduced systemic vascular resistance [26]. Some of these changes may persist beyond pregnancy and may enhance ventricular contractile efficiency. As LV isovolumic contraction time reflects the time required for the ventricle to generate sufficient pressure to open the aortic valve, improved ventricular performance or reduced afterload in parous women could contribute to the shorter IVCT observed in this group.

No statistically significant associations were identified between the cardiovascular parameters measured and the demographic variables of smoking status and ethnicity. As presented in Table 1, the low prevalence of smokers and the limited ethnic diversity within the study population limited the ability to identify any statistically significant differences.

Within the study, there was an increase in left atrial area at embryo transfer compared to the baseline visit for both protocols along with a significant reduction in LV end-diastolic diameter. These observations likely reflect a hyperdynamic, volume-loaded circulation. As both assessments, Visit 1 and Visit 2, were performed during the luteal phase, this change is unlikely to reflect the cycle phase alone and may instead relate to progesterone supplementation used in the medicated and natural modified FET cycles. Studies have provided evidence demonstrating that progesterone, independent of oestrogen, increases plasma volume and extracellular fluid volume, increasing cardiac preload and leading to greater left atrial filling and transient enlargement in atrial dimensions [27]. This pattern closely resembles early pregnancy-like cardiovascular adaptation, which is also progesterone-dominant [28,29]. Whilst the left atrial area increases to accommodate the increased pre-load, studies in early pregnancy have shown an additional increase in LV end-diastolic diameter [30] reflecting physiological cardiac adaptation to increased preload and blood volume during pregnancy. We therefore cannot explain the reduction in LV end-diastolic diameter seen in this study as there is no direct information on the effects of acute progesterone administration on cardiac contractility in humans in the literature.

In women undergoing frozen embryo transfer, both NM and medicated FET cycles demonstrated normal cardiac function. Medicated FET cycles were associated with several indices suggestive of enhanced cardiac performance, including higher septal E′ and S′ velocities and transmitral E-wave velocity, and lower myocardial performance index and shortened isovolumic relaxation time. Furthermore, medicated FET cycles showed improved average global longitudinal strain in Visit 2 compared to Visit 1, suggestive of better preserved longitudinal myocardial deformation, as opposed to women in NM FET cycles who demonstrated slightly worse strain. However, apart from average global longitudinal strain, all pre-mentioned parameters, including septal E′ and S′ velocities, transmitral E-wave velocity, myocardial performance index and isovolumic relaxation time, were noted to be significantly different at both Visits 1 and 2, suggesting that these parameters may reflect pre-existing physiological variation rather than effects attributable to the FET protocol. Given that the two groups had similar demographic characteristics and blood pressure, and the fact that women in NM FET cycles were predominantly White, the difference between these parameters of LV systolic and diastolic function cannot be attributed to background population differences and must be an accidental finding. As this was an observational study, self-selection of the treatment protocol and other influencing factors may have contributed to the findings—variables that would typically be controlled in a randomised study. Despite these baseline differences, the observation of more negative global longitudinal strain between the two visits in medicated cycles remains notable. The less negative strain observed in natural modified cycles is consistent with the subtle, subclinical impairment in longitudinal systolic function, aligning with the reductions in tissue Doppler velocities and increased myocardial performance index. Given that longitudinal strain is sensitive to changes in loading conditions [26] and myocardial relaxation [27], these findings support a functional, protocol-related effect rather than intrinsic myocardial pathology. It is likely that the exposure to oestrogen in the medicated FET group influences vascular tone [31] and myocardial relaxation, leading to the improved LV strain. This mechanism is supported by substantial evidence demonstrating that oestrogen exerts both rapid and longer-term effects on the cardiovascular system through multiple pathways [32]. Furthermore, Donno et al. demonstrated that pregnancies following medicated FET cycles show significantly lower first-trimester uterine artery pulsatility index when compared to natural FET cycles, which they hypothesise to be due to a hyperoestrogenic state from exogenous oestrogen administration until 10 weeks gestation [33]. As uterine artery Doppler indices have been shown to correlate directly with maternal systemic vascular resistance and central haemodynamics [34], this finding further substantiates the theory that exogenous oestrogen in medicated FET cycles induces systemic vasodilation, favourably altering cardiac loading conditions, thereby explaining the improved LV strain observed in our study.

### 4.2. Strengths and Limitations

The study’s strengths lie in its prospective design and the standardised methodology used for cardiovascular assessments. Furthermore, all echocardiographic assessments were performed by a select few experienced sonographers with specialised training in cardiac imaging, reducing the likelihood of measurement bias and inter-operator variability.

Considering the limitations of the study, given the lack of previous research assessing cardiac changes in FET cycles, no power calculation could be performed. Furthermore, this was a relatively small study cohort, particularly within the natural/natural modified FET group, which included only 13 participants. As a result, this study may have been underpowered to detect smaller differences between groups, and therefore, the findings should be confirmed through larger studies in the future. We also note that with this observational study, there is a degree of variability within the medications and dosing between patients based on individual response within the protocols. Given that study participation was voluntary, the potential for selection bias, such as volunteer bias, cannot be excluded and may limit the generalizability of the findings. Although common modifiable and non-modifiable cardiovascular risk factors were accounted for, several important variables, including hypercholesterolemia, dietary habits, alcohol consumption, and family history, were not evaluated or adjusted for and should therefore be considered when interpreting the results.

## 5. Conclusions

FET cycles are overall associated with an increase in left atrial area, most likely as a result of preload due to progesterone administration, and, in medicated cycles, with an improvement in LV average global longitudinal strain following sustained oestrogen administration. Further longitudinal studies are required to clarify the persistence of these findings and their potential implications for cardiovascular adaptation and pregnancy-related outcomes.

## Data Availability

The datasets presented in this article are not readily available due to restrictions from the Human Fertilisation and Embryology Authority (HFEA).

## Supplementary Materials

The following supporting information can be downloaded at: https://www.mdpi.com/article/10.3390/jcm15124717/s1, Table S1: Cardiovascular parameters presented for the total population and sub-categorised according to treatment protocol. *p*-value < 0.05 in Student’s *t*-test is considered statistically significant. Table S2: Multilevel linear mixed-effects models for cardiovascular parameters. The table demonstrates *p*-values for the fixed effects of the full model. Table S3: Multilevel linear mixed-effects models for cardiovascular parameters. The table demonstrates the coefficients (95% CI) for the fixed effects of the final model including only statistically significant variables. The initial model with all variables is presented in Table S2. Supplementary Material S1: Description outlining the echocardiography assessment methodology.
