# Supplementary material for: Cardiovascular Changes in Women Undergoing Medicated and Natural Frozen Embryo Transfer Cycles: A Prospective Observational Cohort Study
Source: J Clin Med. 2026 Jun 17;15(12):4717. doi: 10.3390/jcm15124717 (PMC13301455; doi:10.3390/jcm15124717)
Supplement: Supplementary file 1 [file jcm-15-04717-s001.zip › jcm-4333745-supplementary.pdf]

Supplementary Files

Supplementary Table S1: Cardiovascular parameters presented for the total population and sub-categorised according to treatment protocol. P-value <0.05 in the Student's t-test is considered statistically significant.

**Variable**	**All subjects (n=72)**	**Natural modified FET (n=13)**	**Medicated FET (n=59)**	**p-value**
**Cardiovascular parameters (mean +/- SD)**				
Haemodynamic variables				
Mean arterial pressure (mmHg)	87.2 ±9.37	85.97 ±8.17	87.48 ±9.65	0.80
LV stroke volume (mL)	74.96 ±20.47	76.12 ±24.63	74.70 ±19.64	0.85
Heart rate (b/min)	66.38 ±10.44	64.69 ±10.81	66.75 ±10.42	0.58
LV cardiac output (L/min)	4.86 ±1.44	4.96 ±2.01	4.84 ±1.30	0.80
Left ventricular systolic function				
Biplane LV end-diastolic volume (mL)	71.84 ±12.26	69.62 ±9.60	72.36 ±12.81	0.60
Biplane LV end-systolic volume (mL)	27.26 ±4.47	26.03 ±4.42	27.54 ±4.47	0.31
Biplane LV ejection fraction (%)	62.53 ±7.79	64.86 ±8.05	61.99 ±7.70	0.26
LV isovolumic contraction time (ms)	60.17 ±15.70	65.54 ±20.19	58.95 ±14.43	0.43
LV ejection time (ms)	296.40 ±28.68	291.77 ±25.24	297.46 ±29.52	0.41
LV myocardial performance index	0.43 ±0.11	0.50 ±0.13	0.41 ±0.09	0.05
Left atrial area (cm^2)	10.85 ±1.94	11.40 ±1.93	10.73 ±1.94	0.31

Leftventricular diastolic function				
Mitral valve E-wave maximum velocity (cm/s)	81.73 ±15.92	76.85 ±17.43	82.81 ±15.52	0.21
Mitral valve A-wave maximum velocity (cm/s)	44.79 ±14.12	45.68 ±15.31	44.60 ±13.98	0.61
Mitral valve E/A-wave ratio	1.98±0.66	1.92±0.62	1.96±0.62	0.77
Mitral valve e-lateral velocity (cm/s)	14.71 ±3.28	13.47 ±3.57	14.99 ±3.18	0.20
Mitral valve a-lateral velocity (cm/s)	9.70 ±2.55	8.76 ±2.24	9.91 ±2.58	0.17
Mitral valve s-lateral velocity (cm/s)	10.06 ±2.63	9.72 ±3.54	10.14 ±2.41	0.43
Mitral valve e-septal velocity (cm/s)	11.88 ±2.32	10.21 ±1.79	12.25 ±2.27	**<0.01**
Mitral valve a-septal velocity (cm/s)	9.04 ±2.01	8.61 ±1.67	9.14 ±2.07	0.49
Mitral valve s-septal velocity (cm/s)	9.24 ±1.77	8.67 ±1.33	9.36 ±1.84	0.13
LV isovolumic relaxation time (ms)	67.6 ±13.9	78.40 ±12.00	65.10 ±13.10	**<0.001**
Left ventricular m-mode				
LV intraventricular septum in diastole (mm)	10.04 ±1.86	10.32 ±1.60	9.98 ±1.92	0.43
LV end-diastolic diameter (mm)	42.17 ±4.48	43.53 ±4.22	41.87 ±4.52	0.18
LV posterior wall in diastole (mm)	9.25 ±1.66	9.11 ±1.55	9.29 ±1.70	0.86
LV end-systolic diameter (mm)	26.11 ±3.34	24.99 ±2.19	26.36 ±3.51	0.15
LV mass (g)	114.49 ±22.57	115.77 ±19.68	114.20 ±23.30	0.65
Global and circumferential strain				
Average global longitudinal strain (%)	-22.85 ±2.76	-22.89 ±3.36	-22.84 ±2.63	0.85

FET: Frozen embryo transfer, SD: standard deviation, LV: left-ventricular.

Supplementary Table S2: Multilevel linear mixed-effects models for cardiovascular parameters. The table demonstrates p-values for the fixed effects for the full model.

**Variable**	Age	Body Mass Index	Ethnicity	Smoking	Nulliparous	Visit	Intended treatment	Interaction – treatment*visit
**Cardiac function variables**								
**Haemodynamic variables**								
Mean arterial pressure (mmHg)	0.293	**0.002**	0.801	0.671	**0.044**	0.379	0.489	0.249
LV stroke volume (ml)	0.835	0.015	0.166	0.470	0.967	0.283	0.395	0.523
Heart rate (b/min)	0.251	0.395	0.434	0.751	0.893	0.355	0.959	0.867
LV cardiac output (L/min)	0.255	**<0.001**	0.340	0.474	0.574	0.504	0.174	0.711
**Left ventricular systolic function**								
Biplane LV end-diastolic volume (mls)	0.576	**0.042**	0.131	0.928	0.825	0.488	0.699	0.491
Biplane LV end-systolic volume (ml)	0.604	**0.031**	0.705	0.289	0.595	0.274	0.761	0.267
Biplane LV ejection fraction (%)	0.360	0.776	0.572	**0.035**	0.178	0.774	**0.036**	0.622
LV Isovolumic contraction time (ms)	0.946	0.340	0.947	0.779	**0.036**	0.941	0.392	0.129
LV ejection time (ms)	**0.002**	0.719	0.068	0.118	0.712	0.892	0.106	0.930
LV myocardial performance index	0.084	0.260	0.250	0.878	0.262	0.756	**0.001**	0.166
Left atrial area (cm^2)	0.364	0.437	0.150	0.313	0.066	0.268	0.579	0.050
**Left ventricular diastolic function**								
Mitral valve E-wave maximum velocity (cm/s)	0.549	**0.039**	0.754	0.410	0.325	0.849	0.128	0.789
Mitral valve A-wave maximum velocity (cm/s)	0.559	0.076	0.614	0.763	0.772	0.829	0.879	0.370
Mitral valve E/A-wave ratio	0.258	**0.002**	0.474	0.258	0.979	0.490	0.167	0.478
Mitral valve e-lateral velocity (cm/s)	0.053	0.839	0.153	0.537	0.889	0.877	0.163	0.948
Mitral valve a-lateral velocity (cm/s)	0.219	0.038	0.507	0.693	0.570	0.200	0.927	0.056

Mitral valve s-lateral velocity (cm/s)	0.299	0.984	0.090	0.405	0.782	0.885	0.957	0.472
Mitral valve e-septal velocity (cm/s)	**0.015**	0.198	0.068	0.863	0.715	0.235	**0.011**	0.800
Mitral valve a-septal velocity (cm/s)	0.339	0.090	0.525	0.314	0.805	0.143	0.383	0.773
Mitral valve s-septal velocity (cm/s)	0.767	0.353	0.222	0.159	0.520	0.810	**0.046**	0.477
Isovolumic relaxation time (ms)	0.967	0.199	0.721	0.175	0.648	0.833	**<0.001**	0.222
**Left ventricular m-mode**								
LV intraventricular septum in diastole (mm)	0.948	0.305	0.394	0.253	0.759	0.965	0.336	0.801
LV end-diastolic diameter (mm)	0.576	**0.042**	0.131	0.928	0.825	0.488	0.699	0.491
LV posterior wall in diastole (mm)	0.912	0.252	0.638	0.996	0.503	0.473	0.853	0.975
LV end-systolic diameter (mm)	0.604	0.031	0.705	0.289	0.595	0.274	0.761	0.267
LV mass (gr)	0.970	0.027	0.606	0.492	0.831	0.577	0.512	0.891
**Global and Circumferential strain**								
Average global longitudinal strain (%)	0.717	0.051	0.067	0.333	0.748	0.142	**0.008**	**0.024**

LV: left ventricular

Supplementary material S1: Description outlining the echocardiography assessment methodology

Using the cross-sectional area of the left-ventricular (LV) outflow tract and the velocity time integral of the pulsed Doppler subaortic waveform, which was recorded in the five-chamber view, stroke volume (SV) was calculated. Following this, cardiac output could be determined by multiplying the heart rate with stroke volume. The following equation was then used to calculate peripheral vascular resistance (PVR): MAP × 80/cardiac output.

Two-dimensional guided M-mode was used to assess LV long axis function using the apical four-chamber view with the septal and lateral sides of the mitral valve annulus. The apical four-chamber view was used, in addition, to assess LV filling dynamics and thereby evaluate diastolic function. Transmitral flow was determined with the sample volume positioned level with the tips of the mitral leaflets when in diastole in the fully open position. The peak E:A ratio was calculated from the peak velocity of late atrial (A) and early atrial (E) filling.

We determined the mitral closing-to-opening time (a) at LV ejection time (b) from start to end of the Doppler subaortic waveform pattern. We then measured the period between closing and opening of the mitral valve. The Tei index was calculated as (a − b)/b.

Doppler tissue imaging was performed using a 3.5 mm sample volume at the septal aspect and 5 mm volume for the lateral aspect of the mitral annulus acquired in the four-chamber view [22]. The peak velocity of early (E′) and late (A′) diastolic filling and peak systolic velocity (S′) were obtained. Both isovolumetric relaxation and contraction times (IVRT/IVCT) were assessed at both septal and lateral sites. The transmitral E:E′ ratio was determined for the septal and lateral margins of the mitral annulus, which has been demonstrated to reflect both pulmonary capillary wedge pressure and left-atrial pressure. 2D speckle tracking echocardiography was performed based on the consensus document of the European Association of Cardiovascular Imaging/America Society of Echocardiography/Industry Task Force to standardize deformation imaging (1).

[21] Badano LP, Kolias TJ, Muraru D, Abraham TP, Aurigemma G, Edvardsen T, et al. Standardization of left atrial, right ventricular, and right atrial deformation imaging using two-dimensional speckle tracking echocardiography: a consensus document of the EACVI/ASE/Industry Task Force to standardize deformation imaging. European Heart Journal - Cardiovascular Imaging. 2018;19(6):591-600.

Supplementary Table S3: Multilevel linear mixed-effects models for cardiovascular parameters. The table demonstrates the coefficients (95% CI) for the fixed effects for the final model including only statistically significant variables. The initial model with all variables is presented in Supplementary Table S2.

**Variable**	Age	Body Mass Index	Ethnicity	Smoking	Nulliparous	Visit	Intended treatment	Interaction – treatment*visit
**Cardiac function variables**								
**Haemodynamic variables**								
Mean arterial pressure (mmHg)		0.75 (0.23, 1.26) $^{++}$						
LV stroke volume (mls)		1.02 (0.08, 1.97) $^{+}$						
Heart rate (b/min)								
LV cardiac output (L/min)		0.11 (0.05, 0.17) $^{+++}$						
**Left ventricular systolic function**								
Biplane LV end-diastolic volume (mls)		0.80 (0.09, 1.51) $^{+}$						
Biplane LV end-systolic volume (mls)								
Biplane LV ejection fraction (%)								
LV Isovolumic contraction time (ms)					-5.82 (-11.29, -0.35) $^{+}$			
LV ejection time (ms)	2.24 (0.93, 3.55) $^{++}$							
LV myocardial performance index	-0.005 (-0.01, -0.001) $^{+}$						0.06 (0.02-0.11) $^{++}$	
Left atrial area (cm^2)						-0.98 (-1.63, -0.33)		

**Left ventricular diastolic function**								
Mitral valve E-wave maximum velocity (cm/s)		-0.87 (-1.56, -0.17) +					-7.76 (-14.91, -0.60) +	
Mitral valve A-wave maximum velocity (cm/s)		0.65 (0.01, 1.29) +						
Mitral valve E/A-wave ratio		-0.45 (-0.07, -0.02) ++						
Mitral valve e-lateral velocity (cm/s)								
Mitral valve a-lateral velocity (cm/s)		0.14 (0.03, 0.24) +						
Mitral valve s-lateral velocity (cm/s)								
Mitral valve e-septal velocity (cm/s)	-0.16 (-0.28, -0.03) +						-1.76 (-2.90, -0.61) ++	
Mitral valve a-septal velocity (cm/s)								
Mitral valve s-septal velocity (cm/s)							-0.95 (-1.83, -0.07) +	
Isovolumic relaxation time (ms)							13.23 (7.44, 19.01) +++	
**Left ventricular m-mode**								
LV intraventricular septum in diastole (mm)								
LV end-diastolic diameter (mm)						3.37 (2.01, 4.73) +++		
LV posterior wall in diastole (mm)								
LV end-systolic diameter (mm)					1.72 (0.23, 3.20) +			
LV mass (gr)		1.44 (0.29, 2.60) +						
**Global and Circumferential strain**								
Average global longitudinal strain (%)		0.12 (0.01, 0.24) +						-2.24 (-4.17, -0.31) +

P= <0.05 +, P = <0.01 ++, P= <0.001 +++

LV: left ventricular
